# How Do the Determinants of Collaborative Consumption Influence Its Use in Healthcare? A Managerial Perspective

**DOI:** 10.34172/ijhpm.8453

**Published:** 2024-11-18

**Authors:** Luigi Piper, Lucrezia Maria de Cosmo, Marco Benvenuto, Carmine Viola

**Affiliations:** ^1^Department of Economics, University of Salento, Lecce, Italy.; ^2^Department of Economics, Management and Business Law, University of Bari, Bari, Italy.

**Keywords:** Collaborative Consumption, Healthcare, Behavioral Beliefs, Normative Beliefs, Control Beliefs, Digital Health Literacy

## Abstract

**Background:**

The primary objective of this investigation is to scrutinize the underlying motivations that may prompt those responsible for health to adopt models of collaborative consumption (CC) as business innovation. Furthermore, the study seeks to assess the congruence of determinants influencing the intention to utilize CC in healthcare, comparing perspectives between responsible for health and digital health consumers.

**Methods:**

Two studies based on the Theory of Planned Behavior (TPB) have been conducted. Study 1 uses a qualitative approach to analyze the determinants in use CC in healthcare of responsible for health of the Italian’s National Health Service. Study 2 uses a quantitative approach to analyze a sample of healthcare consumers, their salient beliefs, digital health literacy, and perceived own health status in determining the intention to use CC in healthcare.

**Results:**

Responsible for health recognize both the benefits, like improved efficiency, and the drawbacks, such as digital illiteracy and privacy concerns. Consumer data reveals that attitudes, social norms, perceived control, and digital literacy significantly influence the intention to use CC in healthcare, with education and age being moderating factors, whereas income is not impactful.

**Conclusion:**

The research ends with a discussion of these findings and their strategic implications for managing decision support systems in healthcare. The research highlights the need for innovation-based strategies in the health system, proposing a new socio-technical health domain to improve management through a participatory approach. The approach emphasizes business innovation, service quality, and cost-efficiency. Finally, the research addresses the gaps highlighted in CC in healthcare adoption, underscoring public-private collaboration and practical strategies for sustainable success.

## Background

**Key Messages**

**Implications for policy makers**

- Responsible for health makers at all levels highlight the economic, organizational/managerial and social benefits of using the collaborative consumption (CC) business model in the healthcare sector.
- Responsible for health at all levels highlight the cultural, technological and security benefits of using the CC business model in the healthcare sector.
- Responsible for health at all levels highlight both the aggregative/organizational benefits linked to greater political cohesion and the professional benefits linked to training and simplification of procedures.
- Responsible for health at all levels highlight the strategic benefits linked to the implementation of simplified access to services for better compliance with the National Health Record.

**Implications for the public**

The results of the study highlight how the adoption of collaborative consumption (CC) in the healthcare sector brings significant benefits for patients, significantly improving their treatment experience. Thanks to advanced digital platforms, patients enjoy easier and faster access to healthcare services, reducing waiting times and associated costs. The ability to interact directly with healthcare professionals and access shared information and resources improves the effectiveness of diagnosis and treatment, allowing for more proactive management of one’s health. Innovative technologies such as internet of things, artificial intelligence (AI), and machine learning enable constant monitoring of health conditions, offering patients personalized and remote care, ideal for those who live in remote areas or have difficulty traveling. This collaborative model increases transparency and patient involvement in the care process, promoting greater satisfaction and better health outcomes.

The use of information technologies has enabled the growth of online platforms that promote user-generated content, sharing, and collaboration,^1,2^ leading to the emergence of new forms of consumption such as collaborative consumption (CC), which is studied under the broader concept of the sharing economy.^3,4^ Specifically, CC has been defined as an economic model based on the shared consumption of goods and services through online platforms^5^ and temporary access to a tangible/intangible resource, with payment linked to market mechanisms.^6,7^ Through CC, people coordinate the acquisition or distribution of a resource through peer-to-peer networks^8^ and without transferring ownership,^9^ with the aim of reducing waste from a sustainable consumption^10^ or anti-consumption^11^ perspective thanks to the use of resources that are often underutilized.

 Research on CC models has mainly focused on the tourism sector,^12,13^ on the car rental,^14^ and on the apparel rental,^15^ neglecting other sectors where the emergence of technology is leading to industrial convergence, paving the way for collaborative and shared activities. In particular, in response to the global COVID-19 crisis, a marked convergence is observed among sectors including healthcare, medicine, engineering, and technology,^16,17^ resulting in a paradigm shift from traditional approaches to novel business dynamics. The pandemic has notably accelerated the integration of technology within healthcare organizations, catalyzing substantial transformations.^18^ Specifically, recent years have witnessed a digital transformation that not only facilitated the emergence of new markets but also spurred innovative business models within the healthcare sector.^19^ In Europe, the European Parliament, through Council Regulation 2021/522 of March 24, 2021, establishes a program of Union action in the field of health for the period 2021-2027. This Regulation provides an instrument to promote actions that can contribute to strengthening the exchange of best practices between Member States in terms of (1) supporting networks for knowledge exchange or mutual learning; (2) addressing cross-border health threats by reducing the risks of such threats and mitigating the consequences; (3) addressing certain internal market issues by proposing high-quality actions that exploit the potential of healthcare innovation and efficiency improvement, avoiding duplication, and optimizing the use of financial resources. The program should also support capacity-building activities related to strategic planning, access to multiple funding sources and investment in interventions, and program implementation. Finally, the program supports the generation of real-world and clinical data to enable the development, approval, evaluation of, and access to effective innovative medicines, including generics and biosimilars, medical devices, and therapies. Overall, through this regulation, the European Community is promoting digital transformation and the use of platforms, such as CC platforms, to monitor and collect information by fostering interoperability especially in the healthcare sector.^20-22^

 CC represents an opportunity for healthcare (hereafter CC in healthcare), especially for physicians, medical organizations, and patients^23^ using digital platforms and platform providers.^24^ Indeed, industrial convergence is leading to the creation of an economic ecosystem based on networks and alliances between physicians and patients and the sharing of resources, such as medical devices, with implications for the process of co-creation of value,^25^ in which the patient plays an active role thanks to the support of technologies^26^ that allow patients themselves to collaborate with healthcare professionals, hospitals, insurance companies, and institutions.^27^ However, the active role of the patient is strongly influenced by Digital health literacy, defined as a set of cognitive and technical skills required to access, understand, and use healthcare information via information and communication technologies.^28^

 Despite these new opportunities for value co-creation, there are few studies on models of CC in healthcare.^29^ Therefore, this research could serve as an attempt to thoroughly analyze the factors that determine effective consumer-public co-creation. Given the opportunity for growth, it is necessary to analyze the factors that may contribute to the development of this form of collaboration in the healthcare sector. The literature has highlighted the determinants of CC,^7,8,30^ but these may vary by context.^31^ This research aims to fill a significant gap in the scientific literature by offering new insights into the adoption and development of business innovation through CC in healthcare. Rather than examining the antecedents of attitude (used as a proxy), this research employs Ajzen’s model^32^ to determine and compare the motivations that drive responsible for health in a healthcare system to use models of CC in healthcare in the context of the Italian National Health System (NHS). This approach sheds light on the collaboration between the public and private sectors in the provision of shared health technological services. It aims to enhance the understanding of the determinants that influence the Intention to use CC in healthcare, thereby enriching the theoretical framework. Ajzen’s model^32^ contributes to addressing this gap by elucidating these determinants, as highlighted by Ashaduzzaman et al^33^ and Lindblom and Lindblom.^34^ After conducting an analysis of the literature, utilizing Ajzen’s model^32^ of the Theory of Planned Behavior (TPB) both a qualitative study, aimed at analyzing determinants from the perspective of responsible for health, and a quantitative study, intended to measure determinants of the Intention to use CC in healthcare by digital health consumers, will be undertaken. Discussion and implications will ensue based on the findings obtained. The outcomes of this research would contribute to the current body of literature by providing insights that can guide both healthcare operators and users in comprehending the motivations for employing CC in this particular context.

## Theoretical Background

###  Collaborative Consumption

 The collective collaborative movement has inspired business innovation by means of decentralizing production, democratizing consumption, and redistributing goods, as outlined by Botsman and Rogers.^35^ This transformative process involves a triadic activity wherein a platform provider facilitates the connection between a consumer seeking temporary resource utilization and a peer service provider who offers access to those resources as a primary service.^7,36^ Operating on a *peer-to-peer* basis, CC manifests as a lateral relationship, wherein the service provider and the customer exist on an equivalent level.^37^

 One model of CC in healthcare is that of e-health,^38^ which, through technological platforms offered by providers that allow communication between doctor and patient, allows diseases to be prevented, diagnosed, and monitored much more effectively, given the growing number of chronic diseases such as diabetes or Alzheimer’s disease and the aging of the population.^39^ E-health also enables the application of artificial intelligence (AI)-based solutions that help improve people’s health and provide proactive health interventions by extending the patient journey beyond hospitals.^40^ The development of these models of CC in healthcare is leading to a paradigm shift in patient care with healthcare that enables continuous monitoring of the patient’s health, even remotely, thanks to the support of the most advanced technologies such as internet of things, AI, machine learning, as well as advanced analytics capacity thanks to Big Data.^41^ In addition, technology-enabled collaboration within healthcare organizations such as hospitals facilitates the transfer of patient records between healthcare professionals, as well as the search for specific medical devices that can be used for patient care, impacting shared value creation, which is perceived as utility value rather than exchange value.^42^

###  The Theory of Planned Behavior/Motivations

 The theoretical basis of the research is Ajzen’s TPB,^32^ which explains the determinants of behavioral intentions or the links between behavioral beliefs, attitudes, norms, and behavioral intentions. According to the theory of self-determination,^43^ the motivations that lead to attitude and behavior are divided into intrinsic and extrinsic motivations. Intrinsic motivations refer to the pleasure derived from the activity itself and the value derived from acting in accordance with the rules, ie, appropriately.^44^ Extrinsic motivations refer to external influences, such as prestige and economic benefits. Within studies of CC, some scholars identify intrinsic motivations associated with pleasure and sustainability, and extrinsic motivations associated instead with economic benefits and reputation.^8^ Pleasure is related to the nature of the activity, which thus aims at pleasure,^8,43,44^ influencing attitudes and behavioral intentions such as word of mouth in some CC environments.^45^ This motivation is very evident in social commerce environments, as evidenced by existing research on social shopping,^46^ where members of the CC network have a sense of belonging to the community with which they interact coupled with an innate desire to establish and maintain relationships with others based on enjoyment.^47,48^ Therefore, the motivation associated with entertainment is actually a search for social benefits derived from sharing user-generated content^49^ and building communities and developing social capital following the emergence and development of Web 2.0.^50^

 Motivations related to sustainability, on the other hand, are intrinsic motivations related to seeking environmental benefits by participating in CC,^51^ or motivations based on environmental and ethical values that may influence attitudes toward product use. For example, environmental savings are important determinants of carsharing behavior.^52^ According to Tussyadiah,^53^ perceptions of environmental benefits and concerns about sustainability have been shown to be important factors in driving consumers to use peer-to-peer accommodations. Hamari et al^8^ state that an individual’s environmental concerns positively influence their propensity to participate in CC, which translates into behavioral intentions. Finally, Roos and Hahn^54^ theorized that CC is driven by economic and regulatory motives, including attention to the value of the biosphere.

 Economic benefits, which tend to be extrinsic motivations, are a clear driver in determining value and behavior.^55^ These are motivations that are driven by an individual and utilitarian interest, such as sharing accommodation among peers,^9,53^ which serves as an incentive to save economic resources.^56^ Participation in sharing therefore represents rational and utility-maximizing behavior on the part of the consumer, who prefers low-cost options within a CC service over exclusive ownership also in terms of future rewards.^57-59^ Thus, economic benefits have a significant and positive impact on attitudes toward CC, which in turn leads to intentions.^8,60^

 Another extrinsic motivation that determines active participation in communities and collaborative activities via online platforms is reputation.^61^ Some studies show that this factor has a strong influence on the decision to share information and knowledge via CC.^62,63^ Very often, reputation, which is determined by word of mouth via recommendations, ratings, and reviews online,^64,65^ is the key element for building trust in communities such as social commerce.^61^ A recent study by Ng^66^ found that trust has a moderating influence on knowledge sharing behaviors and attitudes and plays an important role in the context of CC. Moreover, reputation is associated with the need to receive rewards in the form of higher status within the community CC.^67^ That is, consumers who share information and knowledge through platforms such as social media receive an improvement in status through the products they use.^68^

 To these social, environmental, and economic motivations, some scholars add innovativeness, self-image congruence, and social norms as determinants of CC participation behavior.^30,69^ Innovativeness is a characteristic of CC actors as they are focused on unique and original activities and approaches.^70^ This is because the CC model provides consumers with a unique form of exchange through procurement mechanisms for goods and services that differ from traditional ones.^71^ Self-image congruence expresses the extent to which a consumer’s identity matches that of a company or brand.^72^ Many studies highlight that consumers prefer brands they can identify with, ie, brands that reflect their self-image and personality.^73^ However, because peer service providers within CC, which have a triadic nature, are diverse and independent, previous studies show that self-image congruence with actors on the same platform is less certain.^9^ However, Setevens et al^30^ show in a recent work that this identification is possible through the provision of services that are increasingly personalized and adapted to consumers’ needs thanks to the collection of information facilitated by the use of technology. Finally, social norms represent another influencing factor in consumption decisions and therefore reflect the behavior of a person’s reference group, which consists of family, friends, and acquaintances.^74^ That is, consumers who use services from CC tend to engage based on the participation of people with whom they have a strong bond.^75^ These social influencers are important determinants of behavior in online social networks,^47,76^ such as word of mouth.^77^

 In the healthcare sector, a recent study explores how a shared healthcare platform, which provides temporary access to health services via a technology platform, facilitates the co-creation of value by patients and providers.^78^ As in other studies, implementing innovative solutions through a sharing economy approach has been shown to positively impact patients’ perceived value and welfare.^79^ The shared-health platforms, which includes a complex ecosystem of healthcare professionals,^15^ empowers patients to actively contribute to enhancing their lifestyle and well-being.^80^ Moreover, the intention to co-create CC by institutions is closely linked to improved health outcomes and economic performance in health spending by reducing costs and uncertainties. Indeed, the sharing economy broadens the scope of primary healthcare, decreases waiting times, and utilizes medical resources more cost-effectively facilitating the co-creation of value and improving the quality of life for vulnerable patients.^78^

## Methods

 The main objective of the research is to analyze the key factors that, according to the responsible for health of the Italian NHS (Study 1, qualitative) and digital health consumers or potential patients (Study 2, quantitative), may influence the use of CC. For this purpose, a dual research methodology based on the TPB is applied.^32^ TPB is concerned with the influence of cognitive components on behavior and is based on the assumption that intention – indications of a person’s willingness to perform a particular action – is the best predictor of that behavior ie, the clear, observable response in a given situation with respect to a particular goal (eg, purchase).^32^ Intention, in turn, is postulated to be a function of a number of determinants whose importance varies depending on the specific behavior. Specifically, these are (1) Attitudes, defined as “the extent to which a person has a favorable or unfavorable evaluation or appraisal of the behavior in question”; (2) Subjective Norm, defined as “the perceived social pressure to perform or not perform the behavior”; and (3) Perceived Behavioral Control, defined as “the perceived ease or difficulty of performing the behavior.”^32^ Study 1 and Study 2 are interconnected as they both utilize the TPB in a comparative manner: Study 1 employs a qualitative approach to analyze responsible for health, while Study 2 uses a quantitative approach to analyze digital health consumers.

###  Study 1. Qualitative Analysis on Responsible for Health

####  Procedure

 To gauge key factors concerning Italian NHS responsible for health, an exploratory research methodology based on qualitative data has been employed. This approach delves deeply into dimensions that quantitative techniques, such as structured surveys, may not fully capture. Qualitative research, rooted in semi-structured interview featuring open-ended questions, permits the interviewer to probe responses, within the confines of scientific rigor and objectivity. Its aim is to interpret data, emphasizing logical rather than statistical relationships. Among the qualitative survey instruments, interviews using semi-structured questionnaires proved to be the most appropriate for the purposes of this research. Interviews are indeed a traditional form of data collection in qualitative studies.^81,82^

 Echoing prior studies,^83,84^ the sampling method aligned with the study’s objective, ie, to develop theories and concepts rather than to generalize results to a wider population. Therefore, a purposive sampling method, chosen deliberately over a probabilistic one, was employed.^85,86^ This approach is employed by researchers when they aim to identify groups, settings, and individuals where the processes under investigation are most likely to be observed.^87^ It ensured the involvement of all categories of actors in the research implementation and allowed for a diverse range of information sources.

**Figure 1:**
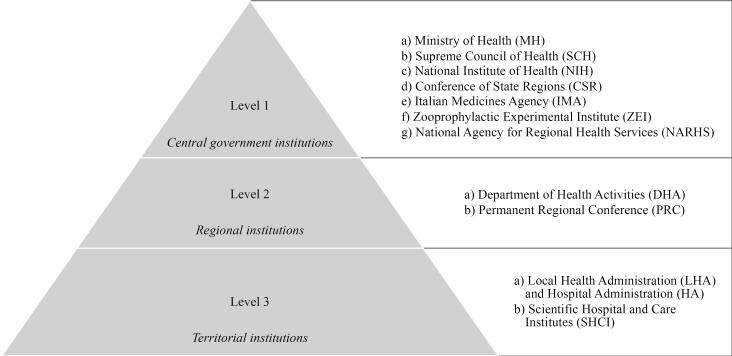
Organizational Model of the Italian National Health Service, According to Law 833/1978 and Law 502/1992.

 The selection of the actors to be interviewed is based on the organizational model of the Italian NHS, according to Law 833/1978 and the Law 502/1992 (Figure 1). The Italian NHS is composed of units and institutions that, according to a pyramid structure, contribute to the achievement of the objectives of health protection of citizens:

- Level 1) Central government institutions: (*a*) Ministry of Health, (*b*) Supreme Council of Health, (*c*) National Institute of Health, (*d*) Conference of State Regions, (*e*) Italian Medicines Agency, (*f*) Zooprophylactic Experimental Institute, and (*g*) National Agency for Regional Health Services.
- Level 2) Regional institutions: (*a*) Department of Health Activities and (*b*) Permanent Regional Conference.
- Level 3) Territorial institutions: (*a*) Local Health Administration and Hospital Administration and (*b*) Scientific Hospital and Care Institutes.

 Each level is assigned tasks, functions, and activities according to a hierarchical logic of administration and territorial authorities. The central government agencies (Level 1) have general planning and resource allocation functions. The regional institutions (Level 2) have legislative, programmatic, and coordinating functions. Finally, territorial institutions (Level 3) have direct administrative management functions.

 Semi-structured interviews have been conducted via telephone by two authors, scheduling appointments of approximately two hours at mutually agreeable times with respondents. All invitees have consented to participate in the research. Prior to commencement, each participant has been provided with a comprehensive overview of the study’s significance. Assurance of anonymity and the anonymization of study outcomes and direct quotations from participants have been reiterated. Subsequently, verbal informed consent has been obtained before proceeding with the interview inquiries.

####  Topics of the Interview

**Figure 2:**
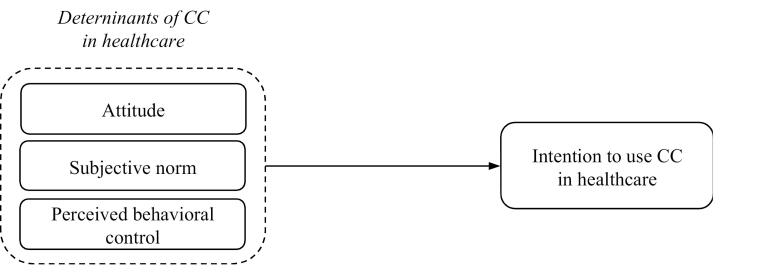
Conceptual Framework of the Qualitative Analysis of Responsible for Health. Abbreviation: CC, collaborative consumption.

 Regarding the topics of the interview, Ajzen’s model^32^ has been followed (Figure 2). This model allows for the highlighting of the following elements: (1) advantages and disadvantages resulting from the use of CC in healthcare (Attitude); (2) potential advocates for using CC in healthcare (Subjective norm); (3) factors or circumstances that could facilitate (incentives) or impede (obstacles) the use of CC in healthcare (Perceived behavioral control). In addition, at the end of the interviews, participants have been asked about additional topics that could potentially influence the use of CC in healthcare.

####  Sample

 Representatives from each level and each institution/body participated in this qualitative study. Specifically, for Level 1, the following responsible for health have been interviewed: 2 representatives of the Ministry of Health, 1 representative of the Supreme Health Council, 2 representatives of the National Institute of Health, 2 representatives of the Conference State Regions, 2 representatives of the Italian Medicines Agency, and 3 representatives of the National Agency for Regional Health Services. For Level 2, the following individuals have been interviewed: 2 representatives of the Department of Health Activities and 2 representatives of the Permanent Regional Conference. Finally, for Level 3, the following have been interviewed: 4 representatives of the Local Health Authorities and 1 representative of the Scientific Hospital and Nursing Institutes. The representatives of Experimental Zooprophylactic Institutes have not been interviewed because the organization in question is not relevant to the research objectives.

####  Results

 The results of the conducted interviews highlight the key factors that may lead the current Italian healthcare system to address and organize the use of CC within the Italian NHS.

 Among the advantages associated with the use of CC in healthcare, the central Level 1 facilities highlight the increase in the quality of the overall service through the standardization of processes and the optimization of resource allocation, the increase in the efficiency of the electronic record and the adaptation of the offer to the demand with a reduction in response times and clinical risk thanks to a necessary change in organizational models (*economic and organizational/management benefits*). The regional entities emphasize, among the benefits derived from the use of CC in healthcare, a better integration between hospital and region, achieved through the sharing of data among all actors and the consequent optimization of diagnosis, treatment, and assistance pathways (*organizational/management and social benefits*). Moreover, the territorial authorities underline the system’s ability to reduce the duration of service provision and costs by shortening waiting lists, improving patients’ quality of life and speeding up the exchange of information between staff and patients (*economic and social advantages*). Regarding disadvantages, all organizations at all levels point to digital illiteracy and, in particular, the potential difficulties in accessing technological services by older people (cultural barriers), the reduction of human contact between operators/physicians and patients, the need to relocate operators, and privacy and cybersecurity issues (technological barriers and security issues).

 The potential advocates for using CC in healthcare appear to be for all facilities at all levels: patients, citizens, pharmaceutical companies, entrepreneurs, healthcare professionals, professional associations (unions), and policy-makers. Unions were also mentioned among those who oppose the use of CC in healthcare, likely for fear of job losses. Rejectors also include older people and, in general, all citizens with little technological experience, which is cited as one of the disadvantages of using CC in healthcare.

 Finally, interviewees indicated the factors favoring the use of CC in healthcare. In particular, all activities performed by organizations that emphasize digital culture in the implementation of National Recovery and Resilience Plan (Piano Nazionale di Ripresa e Resilienza) milestone M1C1-56. While Level 1 entities mention among the facilitators all activities that determine a stronger commitment from responsible for health associated with a greater awareness of the usefulness of CC in the context of the pandemic scenario (aggregate/organizational facilitators as they are associated with greater political cohesion). The Level 2 entities mention training and simplification of processes (facilitations of a professional nature associated with better preparation of technicians). Finally, also the Level 3 entities highlight activities leading to the simplification of access to services, affecting the use of the electronic health record. In contrast, potential barriers to the use of CC in healthcare for organizations at all levels were found to be barriers of cultural nature – such as different procedures based on the user category – barriers of a technical/structural nature – the need to adopt unavailable technological systems – and economic barriers – such as the attribution of a cost to access the service.

Table 1 summarizes the results of the qualitative study in relation to the determinants of CC in healthcare identified by responsible for health in line with the European Digital Health Action Program and the impact of its implementation in the medium to long term.

**Table 1:** Key Factors Leading the Italian Healthcare System to Address the Use of Collaborative Consumption Within the Italian NHS in Line With the European Digital Health Action Program and the Impact of Its Implementation in the Medium to Long-term

**Key Factors**	**Results of Interviews**	**European Action Program on Digital Health**	**Impact**
Advantages	Increased quality of overall service, through standardization of processes to optimize resource allocation and greater attention to electronic records	Measures to achieve the objective Art. 4, letter (a)	Reduction in expected times for healthcare service delivery
	Integration of hospitals and regions resulting in optimization of diagnostic, therapeutic, and care pathways	Measures to achieve the objective Art. 4, letter (f)	Increased quality of care outcomes
	Accelerating the exchange of information in the delivery of services by increasing the exchange of information between health professionals and patients	Measures to achieve the objective Art. 4 (g)	Reduction of passive mobility
Disadvantages	Risk of increasing inequity in access to technology services for older health consumer	Measures to achieve the objective Art. 4 (f)	Hospitalization rate/integrated home care
	Implementation gap in digital services due to the digital divide	Measures to achieve the objective Art. 4 (a)	Training against the digital divide
Favorable subjects	Patients or legal representatives (children and the elderly)	All actions	Increased quality of service
	Pharmaceutical companies	All actions	Increased appropriateness of drug prescribing or Entrepreneurs
	Entrepreneurs	All actions	Increased production capacity of equipment
	Healthcare professionals	All actions	Increased level of safety and quality of service
	Managers	All actions	Increased quality of organizational and individual performance
	Business associations (unions)	All actions	Increased safety
	Policy-makers	All actions	Simplify implementation of health legislation
Incentives	Digital Culture in the implementation of PNRR Milestone M1C1-56	Measures to achieve the objective Art. 4, subparagraph (f)	Spending capacity/digital health integration projects
	Greater simplification of accessibility to health services	Measures to achieve the objective Art. 4, letter (e)	Compliance with the electronic health record
Obstacles	Different procedures based on the user category	Measures to achieve the objective Art. 4, letter (a)	Participation in programmes to digitalize health processes
	Lack of adequate digital infrastructures to ensure connection and interoperability of data in accordance with EU standards, attribution of costs to the service	Measures to achieve the objective Art. 4, letter (a)	Digital infrastructure investment plan
Additional topics	Emergency systems in case of technical problems of the system	Measures to achieve the objective Art. 4, letter (b)	Reduction of maintenance times of the techno-digital system
	IT security and protection of privacy	Measures to achieve the objective Art. 4, letter (e)	Adopt blockchain systems in line with digital evolution

Abbreviations: EU, European Union; IT, information technology; PNRR, Piano Nazionale di Ripresa e Resilienza; NHS, National Health System.

###  Study 2. Quantitative Analysis on Digital Health Consumers

**Figure 3:**
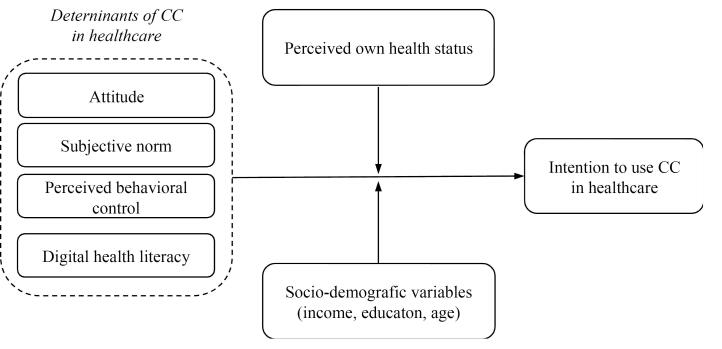
Conceptual Framework of the Quantitative Analysis on Digital Health Consumers. Abbreviation: CC, collaborative consumption.

 To evaluate the determinants of the consumer’s intention to use a CC in healthcare, a quantitative study has been conducted by administering a structured questionnaire, containing the questions identified through a pilot study, to a sample of potential digital health consumers. Usually, research has extended the TPB to incorporate additional predictors able to describe and explain a considerable proportion of the variance in intention or behavior,^88^ and has generally taken into account past behavior.^89^ In this research, conducted in the healthcare sector, it is also expected that the determinants identified in the TPB are significant in shaping the Intention to use CC. However, past behavior is interpreted as Digital health literacy,^90^ which results from a set of cognitive and technical skills developed over time to access, understand, and use information and communication technologies in healthcare. Individuals who are more literate in digital health are more likely to perceive fewer barriers and more advantages in using a CC, thus positively influencing their intention to use it. Additionally, socio-demographic variables (income, education, age, and perceived own health status) are considered as moderating variables. As demonstrated in other studies,^91^ higher income levels can facilitate access to technology, thereby potentially strengthening the relationship between the determinants of the TPB and Intention to use CC in healthcare. In particular, individuals with higher income are likely to experience fewer barriers and perceive more advantages in using digital health technologies, leading to a stronger Intention to use CC solutions. Increased financial resources enable greater access to technology, which in turn reduces technical or economic constraints that could otherwise hinder adoption. As a result, income acts as a significant moderator by enhancing the positive impact of TPB determinants on the intention to engage with CC solutions. Similarly, higher levels of education are generally related to better health literacy and technological skills, improving individuals’ ability to understand and effectively use healthcare technology platforms.^92,93^ Consequently, individuals with higher educational attainment are better equipped to recognize the advantages of using CC solutions, which strengthens the influence of the TPB determinants on their intention to adopt such technologies. In this sense, education serves as a moderator by enhancing the ability to understand and utilize healthcare innovations, thereby amplifying the relationship between key predictors and the intention to engage with CC solutions. Also, age can moderate the relationship between the independent variables and the Intention to use CC in healthcare. As observed in previous studies,^94,95^ younger individuals tend to be more adaptable to emerging technologies compared to older individuals. This greater adaptability allows younger users to interact with CC platforms more effectively, potentially perceiving them as easier to use. However, as age increases, the moderating effect on the relationship between the key determinants of the TPB and the Intention to use CC becomes more complex. Specifically, the moderating effect of age is negative in relation to Attitude. Older individuals may perceive more barriers and exhibit lower perceived usefulness, weakening the effect of attitude on intention. Instead, the moderating effect of age is positive in relation to Subjective norm. Older individuals are more likely to rely on the opinions and recommendations of others, which strengthens the influence of subjective norms on their intention to adopt CC platforms. Finally, individuals who perceive their health as poor may be more motivated to seek out alternative health management solutions, such as CC platforms, to improve their well-being. This heightened motivation increases their receptiveness to the perceived advantages and social influences that promote the use of such technologies. As a result, those with a lower perceived health status are more likely to view CC platforms as valuable tools for managing their health, thus amplifying the effect of TPB determinants on their intention to use these solutions. In contrast, individuals who perceive their health status as good may feel less urgency or need to engage with CC platforms, as they may not see immediate benefits for their current well-being. This perception could weaken the impact of the determinants on their intention to use these platforms. Thus, perceived own health status acts as a negative moderator in this relationship. Thus, the following hypotheses are proposed, and Figure 3 illustrates the conceptual framework of Study 2.

- H1. Attitudes, Subjective norms, Perceived behavioral control, Digital health literacy positively affects Intention to use CC in healthcare.
- H2. Income, Education, Age, and Perceived own health status moderates the relationship between determinants of CC in healthcare and Intention to use CC in healthcare.

####  Pilot Study

 As indicated in Fishbein and Ajzen,^88^ the TPB requires a two-stage research process. A pilot study identifies the salient beliefs of the Intention to use CC in healthcare to be used in a successive main study.

 Following common practice in psychological tests,^88,96-100^ an open-ended questionnaire has been administered to identify the salient beliefs at the basis of intention determinants. In particular: Attitude, by asking them to enumerate the advantages and disadvantages of the use CC in healthcare; Subjective Norms, by asking them to specify possible people or groups of people that might approve or disapprove their decision; Perceived Behavioral Control, by asking them to enumerate facts or circumstances that could facilitate or impede the decision. As regards the Subjective Norms, the answers refer only to subjects in favor of the use of a CC in healthcare.

 Participants for the pilot study have been recruited using convenience sampling, ensuring voluntary and anonymous involvement. Two trained interviewers have administered the questionnaires over a two-week period in October 2023, between 10:00 am and 6:00 pm, at university campuses and shopping districts. Interviewers have randomly approached individuals, explained the procedure, obtained informed consent, and reassured participants of the anonymity and non-evaluative nature of the responses. Participants have then been given a link to a Google Form to complete the questionnaire immediately on their smartphones or tablets.

 The pilot study has been conducted on a sample of 176 subjects – 84 males and 92 females, aged between 18 and 72 years old (mean = 42.1, standard deviation = 12.7). The sample is representative of the Italian population in terms of gender and age.^101^

 The data obtained from the pilot study have been used to select reliable and valid items for inclusion in the final questionnaire. Each set of items intended to directly evaluate a specific construct should exhibit a high level of internal consistency, and the measurements of the different constructs should show clear discriminant validity. To achieve these goals, one or two items may need to be eliminated for each construct. Therefore, in this pilot study, only items with a frequency higher than 20% have been considered. Furthermore, confirmatory factor analysis (CFA) is a method for assessing the quality of the scales to be included. For this reason, a CFA has been conducted in the main study. Results of the pilot study are described in Table S1 (See Supplementary file 1).

####  Main Study

#####  Procedure

 Consistent with methodologies employed in similar research,^102^ participants have been recruited through convenience sampling, ensuring that their involvement was both voluntary and anonymous. Although convenience sampling may introduce potential biases due to its intrinsic lack of representativeness,^103^ special attention has been given to mitigate these biases. By carefully analyzing the sample, efforts have been made to ensure that it accurately reflected the demographic and social characteristics of the broader Italian population. This approach helps in making the findings more generalizable despite the inherent limitations of convenience sampling.

 The questionnaires have been distributed by four trained interviewers over a four-week period, conducted in November 2023 (2 weeks) and May 2024 (2 weeks), between 10:00 am and 6:00 pm. The locations for data collection included public areas such as university campuses and shopping districts. Interviewers have approached potential participants at random, explaining the procedure and obtaining informed consent before asking them to complete the questionnaire. To alleviate any anxiety associated with the evaluation process,^104^ interviewers have reassured participants that there were no right or wrong answers and have emphasized the anonymity of their responses. Participants have also been informed that the collected data would be used solely for scientific and academic purposes. Subsequently, the interviewers have provided participants with a link to a Google Form, enabling them to access and complete the questionnaire immediately via smartphone or tablet.

#####  Questionnaire

 The questionnaire consisted of multi-item measures of the relevant constructs that have been developed from the results of the pilot study (See Table S1) and other constructs and variables included in the model (See Figure 3). More specifically, respondents have been asked (7-point Likert scale) to indicate: (1) the probability that the previously identified advantages/disadvantages might occur in using CC in healthcare and the perceived importance of each advantage/disadvantage; (2) the likelihood that important others would exert an influence on their Intention to use CC in healthcare and the perceived importance attributed to others’ opinions; (3) the probability that the previously identified situations could affect their Intention to use CC in healthcare and the importance they assigned to such situations. Next, respondents have indicated both the strength of their Intention to use CC in healthcare and the likelihood that they would enact such behavior. The questionnaire has included an instrument to measure respondents’ Digital health literacy, ie, the 8-items (7-point Likert scale) eHealth Literacy Scale (also known as “eHEALS”).^90,105^ Finally, the questionnaire has collected social-demographic data (gender, age, educational level, annual income) and Perceived own health status (“Terrible,” “Poor,” “Neither bad nor good,” “Discreet,” and “Optimal”). An attention filter has been included in the questionnaire (“If you read this question, please select 5”). The respondent who answered incorrectly have been excluded from the analysis.

#####  Sample

 The sample consisted of 752 Italian participants (Table S2): 391 men (48%) and 361 women (52%), aged between 18 and 71 years (mean = 41.6 years and standard deviation = 13.3). On the basis of income, the sample is divided as follows: 45.3% have an income below €20 000; 40.4% have an income of between €20 000 and €50 000; 13.0% have an income of between €50 000 and €100 000, and 1.5% have an income higher than €100 000. In addition, the sample consists of 298 (39.6%) of those with a university or higher degree, while the remaining 454 (60.4%) of the sample is made up of those with a high school diploma or lower degree. The selected sample is reflective of the Italian population.^101,106^

#####  Data Analysis

 In order to operationalize Ajzen’s determinants^32^ and Intention to use CC in healthcare, the score given to each determinant have been multiplied by the respective probability, and all these values have been averaged.^96^ Items BB4, BB5, BB6, CB4, CB5, and CB6 have been considered reverse items since the express disadvantage and impediments.

 In the next step, the multi-normality of the data and the presence of common method variance have been evaluated using the Mardia’s test and the common latent factor technique,^104,107^ respectively. A CFA have been performed to validate the measures used. Measurement models utilizing composite indicators can effectively model conceptual variables by combining elements to form a new variable.^108^ These composite indicators provide a convenient method for summarizing data and can measure the properties related to the focal concept, such as attitudes, perceptions, and behavioral intentions.^109,110^ According to Sarstedt et al,^110^ path analysis is unbiased when estimating data from a composite model population. Consequently, the hypothesis was tested using path analysis. Additionally, the analysis considered the potential moderating effect of socio-demographic variables. This has been achieved by creating product terms, which involved multiplying the predictor and the moderator variable.^111-113^ Therefore, a path analysis has been conducted setting attitude, Subjective norm, Perceived behavioral control, and Digital health literacy as the independent variables, Intention to use CC in healthcare as the dependent variable and Level of education, Income, and Perceived own health status as moderators. A one-tailed *P* value of less than.050 have been considered statistically significant. All analyses have been conducted using AMOS version 26 and SPSS version 29.

####  Results

 Preliminary analysis demonstrates that the data distribution is not multivariate normal (Mardia’s multivariate skewness b = 25.67 > 0.00, *P* < .001, multivariate kurtosis b = 2675.98 >1088, *P* < .001), therefore the bootstrapping technique (k = 5000) was adopted to evaluate the model.^114^ The common latent factor test suggests the absence of common method variance bias since the variance extracted by the common factor is 29.7%, lower than the 50% threshold.^115^ The CFA reveals adequate fit statistics (See Tables S3 and S4): Chi-square/degree of freedom (χ^2^/df) = 2.475 < 5.000, *P* < .001; goodness of fit index (GFI) = 0.911 > 0.900; comparative fit index (CFI) = 0.873 > 0.800; normed fit index (NFI) = 0.816 > 0.800; standardized root mean square residual (SRMR) = 0.056 < 0.080.^115-120^ Discriminant validity (See Table S4) is established as the average variance extracted indices surpass the threshold of 0.50. Additionally, for each construct, the square root of the average variance extracted exceeds the correlations between the construct and any other constructs in the model. The measurement model also shows convergent validity, with all factor loadings being above 0.50 and construct reliability values exceeding 0.70, meeting acceptable standards.^115,119^ Furthermore, with all bivariate correlations being below the 0.70 threshold, multicollinearity concerns are mitigated.^121^ Lastly, all Cronbach’s α values are above 0.70, indicating that the scales have adequate internal consistency and reliability.^122^

 Results from a maximum likelihood estimation return acceptable fit statistics (χ^2^/df = 2.579; *P* < .001; GFI = 0.923; CFI = 0.887; SRMR = 0.085). As shown in Table 2, Attitude emerges as a crucial determinant, exhibiting a strong and highly significant positive effect (standardized β= 0.710, standard error [SE] = 0.048; t = 7.008, *P* < .001). This suggests that individuals who hold favorable views about CC are significantly more inclined to intend its use within healthcare settings. Furthermore, Subjective norm also play a pivotal role. The significant effect indicates that the perceived social pressure or influence from others contributes substantially to shaping individuals’ intentions to use CC in healthcare (β= 0.222, SE = 0.051; t = 4.130, *P* < .001). This underscores the importance of social context and the impact of others’ opinions on decision-making processes related to CC in healthcare adoption. Perceived behavioral control is another significant predictor, demonstrating a robust positive and significant relationship with the Intention to use CC in healthcare (β= 0.359, SE = 0.066; t = 29.540, *P* < .001). This finding highlights the importance of individuals’ confidence in their ability to utilize CC in healthcare effectively. When people feel capable and in control of their ability to implement CC in healthcare, they are more likely to intend to use it. Additionally, Digital health literacy shows a significant, albeit smaller, positive effect on Intention to use CC in healthcare (β= 0.077, SE = 0.059; t = 3.179, *P* < .001). This suggests that individuals who possess higher levels of Digital health literacy, which includes the ability to seek, understand, and use digital health information, are more likely to consider using CC in healthcare. While the effect is not as pronounced as other factors, it still highlights the relevance of digital competencies in the adoption of CC in healthcare. These results confirm H1.

**Table 2:** Results of Path Analysis

**Path to Intention to Use CC in Healthcare**	**β**	**SE**	***t***	***P***
Attitudes	0.710	0.048	7.008	.000
Subjective norms	0.222	0.051	4.130	.000
Perceived behavioral control	0.359	0.066	29.540	.000
Digital health literacy	0.077	0.059	3.179	.001
**Interaction Terms**	**β**	**SE**	***t***	***P***
*Attitudes* × Income	0.006	0.070	0.715	.475
*Attitudes* × Level of education	–0.072	0.310	–2.605	.009
*Attitudes* × Age	–0.088	0.062	–9.978	.000
*Attitudes* × Perceived own health status	0.024	0.108	1.285	.199
*Subjective norms* × Income	–0.028	0.128	–1.394	.163
*Subjective norms* × Level of education	–0.144	0.104	–7.948	.000
*Subjective norms* × Age	0.044	0.042	4.939	.000
*Subjective norms* × Perceived own health status	–0.054	0.147	–3.011	.003
*Perceived behavioral control* × Income	0.033	0.191	0.950	.342
*Perceived behavioral control* × Level of education	–0.186	0.045	–4.155	.000
*Perceived behavioral control* × Age	–0.095	0.252	–0.954	.340
*Perceived behavioral control* × Perceived own health status	0.014	0.292	0.548	.583
*Digital health literacy* × Income	–0.007	0.298	–0.246	.805
*Digital health literacy* × Level of education	0.365	0.068	4.997	.000
*Digital health literacy* × Age	0.038	0.300	1.358	.174
*Digital health literacy* × Perceived own health status	–0.103	0.064	–11.502	.000

Abbreviations: CC, collaborative consumption; SE, standard error. Note. β = standardized coefficients; N = 752. P values less than.050 are considered statistically significant.

 The moderated regression analysis reveals several key insights regarding the Intention to use CC in healthcare. Notably, Income does not serve as a moderating factor in the relationship between salient beliefs, Digital health literacy, and the Intention to use CC in healthcare. This is evidenced by the fact that all associated *P *values surpass the significance threshold value of.050, indicating no significant interaction effects involving income and confuting part of H2. The absence of income as a moderating factor in this study may be explained by several factors. One possibility is that, in the specific context of digital health and CC, income may not have the same moderating effect as in other technology-driven contexts. Unlike other areas where higher income directly translates to better access to resources and technology,^91^ digital health services are increasingly designed to be accessible across diverse socio-economic groups. The widespread availability of digital health technologies, particularly through mobile devices and public health platforms, could further diminish the relevance of income as a barrier. Healthcare services may prioritize equity and inclusivity, offering access to low-cost or free digital health tools, which would mitigate the impact of income on the Intention to use CC solutions. This could explain the lack of significant interaction effects between income and the TPB variables, as income no longer plays a critical role in shaping access to or perceptions of healthcare technology.

 In contrast, the Level of education exhibits a significant moderating influence. Specifically, higher levels of education are associated with a diminished effect of attitude (β = –0.072, SE = 0.310; t = –2.605, *P* = .009), Subjective norm (β = –0.144, SE = 0.104; t = –7.948, *P* < .001), and Perceived behavioral control (β = –0.186, SE = 0.045; t = –4.155, *P* < .001) on the Intention to use CC in healthcare. This implies that as educational attainment increases, the positive impact of these factors on the Intention to use CC in healthcare decreases. Conversely, Education positively moderates the effect of Digital health literacy (β= 0.365, SE = 0.068; t = 4.997, *P* < .001), suggesting that individuals with higher educational backgrounds are better able to utilize their Digital health literacy in forming intention to use in CC in healthcare.

 Age also plays a crucial moderating role. The data indicate that older individuals experience a reduced impact of Attitudes on their Intention to use CC in healthcare (β= –0.088, SE = 0.062; t = –9.978, *P* < .001), signifying that positive attitudes towards CC in healthcare translate less effectively into behavioral intention as age increases. However, Age enhances the influence of Subjective norm (β= 0.044, SE = 0.042; t = 4.939, *P* < .001), meaning that older individuals are more susceptible to social pressures in their decision to adopt CC in healthcare. Age does not significantly moderate the effects of Perceived behavioral control and Digital health literacy, as their respective *P *values do not reach statistical significance value of.050.

 Furthermore, Perceived own health status serves as a significant negative moderator for the effects of Subjective norm (β= –0.054, SE = 0.147; t = –3.011, *P* = .003) and Digital health literacy (β= –0.103, SE = 0.064; t = –11.502, *P* < .001) on the Intention to use CC in healthcare. This indicates that individuals who perceive their health status to be better are less influenced by social norms and their Digital health literacy when it comes to forming intentions to use CC in healthcare. However, perceived health status does not significantly affect the relationships involving attitude and Perceived behavioral control, as indicated by non-significant *P* values. These results confirm H2.

## General Discussion

 This research has examined the determinants of the use of CC in healthcare to understand which of them might be consistent among responsible for health and digital health consumers. Two studies have been conducted based on Ajzen’s^32^ model, a qualitative study involving Italian NHS responsible for health and a quantitative study involving digital health consumers.

 The adoption of this approach enabled the identification of the advantages and disadvantages associated with implementing CC in healthcare, achieving results that are consistent with findings observed in comparable studies on the sharing economy.^4^ Moreover, this approach allows for the exploration of facts or circumstances that may support or impede the utilization of CC in healthcare, as well as the examination of issues that could potentially facilitate or hinder its application in the healthcare domain. In addition, the quantitative study has also considered Digital health literacy as one of the determinants of Intention to use CC in healthcare, as well as two moderators: Perceived own health status and socio-demographic variables (income, level of education, and age).

 The results of the qualitative study highlight the alignment of the motivations for the use of CC in healthcare by Italian NHS responsible for health, divided into three levels, with the European Union Program for Action in Health for the period 2021-2027, according to Council Regulation 2021/522 of March 24, 2021. In particular, responsible for health at all levels highlight the economic, organizational/managerial and social benefits of using CC in the health sector, the cultural, technological and safety issues, the facilitations of aggregative/organizational nature related to greater political cohesion, the facilitations of a professional nature related to training and simplification of procedures, the facilitations of an economic nature related to resource scarcity, and finally, the facilitations of a strategic nature related to the implementation of simplified access to services for better compliance with the National Health Record. There are also potential barriers to the use of CC in healthcare that are cultural, related to Digital health literacy, and technical/structural, related to the lack of adequate digital infrastructures to ensure data connectivity and interoperability. Finally, the potential advocates for the use of CC in healthcare for all entities at all levels appear to be patients, citizens, pharmaceutical companies, entrepreneurs, healthcare professionals, professional associations (unions), and policy-makers.

 The quantitative study confirms the importance of attitudes, Subjective norms, and Perceived behavioral control for the Intention to use CC in healthcare. In particular, as shown in Table S1 and confirmed with the CFA in Table S2, the linkages are confirmed by the possibility of making the use of current health services more efficient by saving time and money, facilitating access to services, and improving the quality of health service outcomes. At the same time, results highlight the need to perfect the semantic language of health-related information that can be used by potential digital health consumers and, therefore, could be unsatisfactory in terms of the needs of the digital health consumers themselves, with consequences of offline health organizations with the standard parameters. In addition, there are issues that advocate the use of CC in healthcare in line with the proposals of the European program, in particular: Doctors, family members, friends, etc. Finally, the points of connection are found in relation to the Perceived behavioral control regarding the effectiveness of CC in terms of orientation and access to services through methods easily identifiable by institutional information channels. At the same time, there are issues related to the use of data in accordance with European regulations and the need to facilitate the use of clinical information (not readily available), which could lead to confusion in outcomes, with negative consequences in terms of users sharing false information, which can damage the image of health services.

 In conclusion, the comprehensive findings underscore the significance of identifying the motivations behind adopting CC in healthcare. These motivations are intricately linked to the degree of innovation and the adoption of novel approaches to utilizing healthcare services, as corroborated by pertinent studies conducted by Hofmann et al^69^ and Stevens et al.^30^ Results of the moderated regression analysis demonstrate that the level of income has no effect in moderating the relationship between salient beliefs and intention to use. However, more educated digital health consumers appear to be less influenced by other people (Subjective norms) and appear to make decisions based on their Digital health literacy (See Table 2). Instead, it seems that young people are more influenced by other people (rather than by their own knowledge), even if they give much weight to the advantages and disadvantages deriving from the use of CC in healthcare. Finally, digital health consumers, as their health worsens, rely on their knowledge and on the opinions of other people.

## Implications

###  Theoretical Implications

 This research presents several significant theoretical and managerial implications. From a theoretical perspective, the findings contribute to the existing body of knowledge by providing new insights into the adoption and development of business innovation within the context of CC in healthcare. The research advances our understanding of the determinants influencing the Intention to use CC in healthcare, thereby enriching the theoretical framework and informing future studies in this domain. Ajzen’s model^32^ offers a contribution to fill the gap in the literature with reference to the determinants of Intention to use CC in healthcare.^33,34^ While most previous studies have focused on analyzing the factors that may influence attitude and Intention to use CC in healthcare by end users^123^ or by companies,^124,125^ this research has compared the determinants of intentions between responsible for health and digital health consumers, shedding light on the issue of collaboration between the public and private sectors in the provision of shared technological services.^112^ Although Study 1 have employed a qualitative approach confined to a sample of individuals responsible for health, this research paves the way for a more in-depth examination of consumer-public co-creation studies. For example, through in-depth interviews, future research could focus on identifying possible moderators in the relationship between determinants and intentions to use CC in healthcare, such as trust in institutions associated with reputation.^55^ Furthermore, researchers and managers could identify and study particular healthcare contexts in which CC in healthcare could be more efficient and attractive. Especially in relation to the results of the interviews with responsible for health. Indeed, although this research provides cues for identifying some of these contexts, conducted two studies that are limited to analyzing users in a general context and do not consider other perceptual or attitudinal moderating variables or other personal characteristics as determinants.

 A noteworthy observation is that CC in healthcare enables patients to voluntarily share their health data, addressing a significant challenge in medical research—namely, the collection of personal circumstances and family history. The reluctance of individuals to share information within the healthcare context may constitute a limiting factor for the primary study, an obstacle that has been endeavored to overcome by ensuring utmost protection of privacy and confidentiality of personal information. However, if patients comprehend that such data sharing contributes to their own well-being and that of others, the willingness to provide and share data through CC in healthcare represents a crucial advancement. This practice is poised to enhance future research through the application of AI, facilitating the generation of health and behavior patterns in response to various diseases. Consequently, future research should focus on developing and optimizing models of CC in healthcare that emphasize patient education and trust-building, ensuring that data sharing is perceived as beneficial and secure. Additionally, investigating the ethical and privacy implications of such data sharing within CC in healthcare frameworks will be essential to foster widespread adoption and maximize the potential benefits for healthcare research.

###  Practical Implications

 The practical implications of this research are consistent with the impacts of technological and business innovation on business management. Within this framework, theory furnishes conceptual instruments to comprehend the intricate manner in which individuals collectively employ technology to execute their work. This research underscores the favorable influence of collaboration on business value within a model of CC in healthcare. To amplify this influence, the combined results of the two conducted studies suggest that organizations should articulate transparent policies delineating the terms and conditions of reciprocity. Such transparency serves to forestall opportunistic behavior during exchanges of rewards that present difficulties in both quantification and monetization. Sustainable monetary benefits serve as strong incentives for engaging in CC in healthcare. Organizations that embrace a CC model in healthcare must strategically position themselves to conduct operations that are not only cost-effective but also sustainable. This necessitates the implementation of evidence-based strategies, particularly the integration of asset-sharing practices. Such practices not only help mitigate operational costs but also play a substantial role in reducing waste and promoting environmental preservation, thus innovating traditional methods of service delivery. Moreover, a resilient business model is imperative for achieving sustained success and exerting a positive societal influence. Interactions on innovation and collaboration platforms must generate value that the platform provider can capture through product sales and transaction fees. In non-profit scenarios, revenue sources may vary among platform users. Managers seeking to capture value through this innovation should explore additional platform participants interested in strengthening the platform’s core interactions.

 The value found in the results of the research primarily identify the citizen’s alliance as an added value. CC in healthcare could innovate all the components of the health and well-being system by enhancing the local proximity node, ie, the “last mile” of the wider regional European health networks. This domain envisages the creation and implementation of a system inspired by four integrated sub-systems: the circular format^126^ applied to hospitals, the network of healthcare operators, the network of value-added services, the citizens’ participation.

###  Practical Implications for the Italian National Healthcare System

 Finally, it would be interesting to discuss the practical implications that the results of this research could have on the Italian healthcare system holds significant practical implications, especially in light of the regulatory framework established by Council Regulation 2021/522 and the organizational structure of the Italian NHS outlined in Law 833/1978. The promotion of CC in healthcare would be effective if based on the results of Study 1 with reference to the responsible for healthcare and the results of Study 2 with reference to the digital health consumers. This promotion aligns with the objectives promoted in both the European program of Union action in health and the Italian NHS. By supporting networks for knowledge exchange and addressing cross-border health threats, CC in healthcare can facilitate the sharing of best practices and enhance coordination between Member States and healthcare entities within Italy.^20-22^ This can lead to improved responses to health challenges and more efficient resource allocation. Moreover, the emphasis on healthcare innovation and efficiency improvement inherent in CC adoption resonates with the goals of optimizing the use of financial resources and promoting digital transformation promoted in Italy.^127^ By leveraging CC platforms to monitor and collect information, healthcare organizations can enhance interoperability, streamline processes, and facilitate the development, approval, and access to innovative medicines and therapies.^128^ In practical terms, the adoption of CC can foster a culture of collaboration and innovation within the Italian healthcare system, encouraging the exchange of ideas and best practices among healthcare professionals and organizations. For example, it can enhance patient access to healthcare services by facilitating the sharing of resources and expertise across different levels of the healthcare hierarchy. This can ultimately lead to improvements in patient outcomes and the overall quality of healthcare delivery.

## Conclusion

 The conclusions of this research underscore the necessity of implementing innovation-based intervention strategies in the healthcare sector. These strategies should address the fundamental needs of both citizens and responsible for health within the Italian healthcare system, thereby contributing to the broader European debate. Citizens act as recipients of institutional intervention, health professionals navigate the challenging role of intermediaries between public institutions and the citizens, and institutional representatives play a crucial role as promoters of policies aimed at safeguarding national interests. Therefore, this research highlights the need to include a new socio-technical domain of health, considering the intellectual capital involved in the technological theme of CC in healthcare. This approach demonstrates how defining this domain can enhance healthcare management through a participatory multilevel approach.^129^ The concept of this new socio-technical domain of CC in healthcare represents a business innovation rooted in the last-mile healthcare management model, inspired by the concept of connective intelligence and termed the Last Mile Service Environment.^130^ Implementing this model has the potential to improve the quality and quantity of services provided to citizens, reduce management costs, and promote the integration and participation of vulnerable and disadvantaged groups of health consumers. Operating within a decentralized system, it aims to extend the quality, reliability, continuity, and geographic reach of healthcare services.^131^

## Ethical issues

 Verbal informed consent was obtained from the respondents to the questionnaire for their anonymized information to be published in this article.

## Conflicts of interest

 Authors declare that they have no conflicts of interest.

## Data availability statement

 The data that support the findings of this study are available on request from the corresponding author. The data are not publicly available due to privacy or ethical restrictions.

## Supplementary files

Supplementary file 1 contains Tables S1-S4.
