# Supplementary material for: How Do the Determinants of Collaborative Consumption Influence Its Use in Healthcare? A Managerial Perspective
Source: Int J Health Policy Manag. 2024 Nov 18;13:8453. doi: 10.34172/ijhpm.8453 (PMC11607737; doi:10.34172/ijhpm.8453)
Supplement: Supplementary file 1 — contains Tables S1-S4. [file ijhpm-13-8453-s001.pdf]

Article title: How Do the Determinants of Collaborative Consumption Influence Its Use in Healthcare? A Managerial Perspective

Journal name: International Journal of Health Policy and Management (IJHPM)

Authors' information: Luigi Piper[1], Lucrezia Maria de Cosmo[2]*, Marco Benvenuto[1], Carmine Viola[1]

[1]Department of Economics, University of Salento, Lecce, Italy.

[2]Department of Economics, Management and Business Law, University of Bari, Bari, Italy.

***Correspondence to:** Lucrezia Maria de Cosmo; Email: lucreziamaria.decosmo@uniba.it

Citation: Piper L, de Cosmo LM, Benvenuto M, Viola C. How do the determinants of collaborative consumption influence its use in healthcare? A managerial perspective. Int J Health Policy Manag. 2024;13:8453. doi:10.34172/ijhpm.8453

Supplementary file 1

Table S1. Results of pilot study. Salient beliefs: Attitudes, Subjective norms, and Perceived Behavioral Control of the intention to use CC in healthcare

Attitudes (A)	*N (%)*	*Subjective norms (SN)*	*N (%)*	*Perceived behavioral control (PBC)*	*N (%)*
A1 Save time and money	156(89%)	SN1 My doctors	165(94%)	PBC1 Possibility to access the services at any time and from any device	160(91%)
A2 Facilitates the use of the service	134(76%)	SN2 Family members	152(86%)	PBC2 There is an assistant who can guide me in my consumer experience	145(82%)
A3 It allows you to get a better service	110(63%)	SN3 Friends	142(81%)	PBC3 Institutions advertise CC systems clearly	114(65%)
A4 It doesn't meet my needs	89(51%)	SN4 Other patients	104(59%)	PBC4 The system requests information that I don't have or that is difficult to find	99(56%)
A5 Returns confusing information	81(46%)	SN5 Colleagues	54(31%)	PBC5 The system spreads my sensitive data in an uncontrolled way	95(54%)
A6 Request the intervention of more personnel than necessary	45(26%)	SN6 Health institutions	51(29%)	PBC6 System failure events are disclosed	29(22%)

Notes
N indicates the number of times the factor was mentioned within the sample. % indicates the proportion of respondents who mentioned the factor.
Sample size = 176.

Table S2: Sample characteristics

	Category	*Sample*	*Italian Population*
Gender (%)	Male	48.0	48.7
	Female	52.0	51.3
Age (in years)	Mean	41.6	46.4
	SD	13.28	-
Income (%)	< 20,000 Euro	45.3	49.9
	Between 20,000 and 50,000 Euro	40.4	44.2
	Between 50,000 and 100,000 Euro	13.0	5.0
	> 100,000 Euro	1.5	0.9
Level of Education (%)	University or higher degree	39.8	34.8
	High school or lower degree	60.2	65.2

Table S3. Description of observed variables

	Item	FL	CR	AVE	Cronbach's α
Attitudes (A)			0.91	0.64	0.85
Facilitates the use of the service	A1	0.81			
Save time and money	A2	0.79			
It allows you to get a better service	A3	0.80			
It doesn't meet my needs	A4	0.77			
Returns confusing information	A5	0.90			
Request the intervention of more personnel than necessary	A6	0.71			
Subjective Norms (SN)			0.90	0.59	0.73
My doctors	SN1	0.91			
Family members	SN2	0.83			
Friends	SN3	0.79			
Other patients	SN4	0.90			
Colleagues	SN5	0.78			
Health institutions	SN6	0.68			
Perceived Behavioral Control (PBC)			0.93	0.70	0.87
Possibility to access the services at any time and from any device	PBC1	0.76			
There is an assistant who can guide me in my consumer experience	PBC2	0.87			
Institutions advertise CC systems clearly	PBC3	0.81			
The system requests information that I don't have or that is difficult to find	PBC4	0.89			

	Item	FL	CR	AVE	α
The system spreads my sensitive data in an uncontrolled way	PBC5	0.91			
System failure events are disclosed	PBC6	0.76			

	Item	FL	CR	AVE	*α*
Intention to use CC (IU)			0.88	0.79	0.91
Strength of the intention to use CC in healthcare	IV	0.91			
Likelihood to use CC in healthcare	IE	0.87			
Digital Health Literacy (DHL)			0.94	0.65	0.76
I know WHAT health resources and services are available through digital devices	DHL1	0.90			
I know WHERE to find healthcare resources and services available through digital devices	DHL2	0.78			
I know HOW to find health resources and services available through digital devices	DHL3	0.81			
I know how to use the health information I find through digital devices to answer my questions about disease	DHL4	0.82			
I know how to use the health information I find through digital devices to fight disease	DHL5	0.67			
I have the skills I need to evaluate the health resources I find through digital devices	DHL6	0.71			
I can distinguish high quality healthcare resources from low quality resources available through digital devices	DHL7	0.75			
I feel confident using health information through digital devices to make decisions about my health	DHL8	0.70			

Notes: N = 752; A = Attitude; SN = Subjective Norm; PBC = Perceived Behavioral Control; IU = intention to Use CC; IV = Intention-as-Volition item, IE = Intention-as-Expectation item, DHL = Digital Health Literacy. FL = Standardized factor loading (all significant at a 0.01 level); CR = Construct Reliability; AVE = Average Variance Extracted; α = Cronbach's α.

Table S4: Results of the Confirmatory Factor Analysis (CFA)

Index	*Value*	*Satisfactory level*	*References*
Chi-square/d.f. (χ^2/d.f.)	2.475	< 5,000	Anderson and Gerbing (1988); Bentler and Bonett (1981); Byrne (2010); Fornell and Larcker (1981); Hair *et al*. (2012); Hoe (2008)
Goodness of Fit Index (GFI)	0.911	> 0,900	
Comparative Fit Index (CFI)	0.873	> 0,800	
Normed Fit Index (NFI)	0.816	> 0,800	
Standardized Root Mean Square Residual (SRMR)	0.056	< 0,080	

Table S5: Discriminant validity matrix

	A	*SN*	*PBC*	*IU*	*DHL*	*CR*	*AVE*
A	*.80*					.91	.64
SN	.	*.77*				.90	.59
PBC	.	.76	*.84*			.93	.70
IU	.32	.12	–.05	*.89*		.88	.79
DHL	.27	.03	.09	.22	.81	.94	.65

Notes: N = 752; A = Attitude; SN = Subjective Norm; PBC = Perceived Behavioral Control; IU = Intention to Use CC; DHL = Digital Health Literacy. CR = Construct Reliability; AVE = Average Variance Extracted. The square root of AVE indices for each variable are reported in italics along the diagonal.
